# Ocular Proteomics with Emphasis on Two-Dimensional Gel Electrophoresis and Mass Spectrometry

**DOI:** 10.1007/s12575-009-9019-7

**Published:** 2009-12-24

**Authors:** Nakul Mandal, Steffen Heegaard, Jan Ulrik Prause, Bent Honoré, Henrik Vorum

**Affiliations:** 1 Eye Pathology Section, Department of Neuroscience and Pharmacology, University of Copenhagen, Copenhagen, Denmark; 2 Department of Medical Biochemistry, Aarhus University, Aarhus, Denmark; 3 Department of Ophthalmology, Aalborg Hospital, Aarhus University Hospital, Aalborg, Denmark

**Keywords:** ocular proteomics, sample preparation, 2D-PAGE, 2D-DIGE, mass spectrometry

## Abstract

The intention of this review is to provide an overview of current methodologies employed in the rapidly developing field of ocular proteomics with emphasis on sample preparation, two-dimensional polyacrylamide gel electrophoresis (2D-PAGE) and mass spectrometry (MS). Appropriate sample preparation for the diverse range of cells and tissues of the eye is essential to ensure reliable results. Current methods of protein staining for 2D-PAGE, protein labelling for two-dimensional difference gel electrophoresis, gel-based expression analysis and protein identification by MS are summarised. The uses of gel-free MS-based strategies (MuDPIT, iTRAQ, ICAT and SILAC) are also discussed. Proteomic technologies promise to shed new light onto ocular disease processes that could lead to the discovery of strong novel biomarkers and therapeutic targets useful in many ophthalmic conditions.

## 1. Introduction

Blindness and visual impairment can affect all manner and ages of people. In 2002 the World Health Organisation estimated that more than 161 million people were visually impaired around the world of which 37 million were blind [1,2]. The consequences of blindness are devastating and wide-ranging on both the individual affected and society, leading to problems such as unemployment in the young and social dependency of the elderly [3]. Sight-threatening disorders may be diagnosed and treated too late to avoid significant loss of vision, and some have no effective means of treatment or prevention at all. Ophthalmic proteomic research promises to advance the management of many debilitating ocular disorders [4,5].

Although the human genome has now been sequenced [6-8] and a number of genes have been discovered that are linked with diseases throughout the eye, a cell's biological state is ultimately dictated by proteins, the end products of the genes [9,10]. However, it is the understanding of how an estimated one million human proteins are encoded by approximately 25,000 genes that is far from clear. Most genes are subjected to alternative splicing following transcription resulting in the production of several transcripts from each gene [10,11]. Furthermore, there appears to exist a poor correlation between the RNA transcript level and the abundance of the encoded protein, suggesting post-transcriptional regulation [12,13]. Finally, the proteins themselves can undergo post-translational modification (PTM) which can significantly alter their function. In effect, the relatively static genome can give rise to an infinite number of dynamic proteomes, and it is this proteome that alters with cell type and state, which governs the phenotype at any particular moment in time [11,14].

Proteomics is able to qualitatively and quantitatively compare protein profiles under different conditions to further explain biological systems and identify the disease markers that promise to become the diagnostic and pharmaceutical targets of the future [4,5]. Two-dimensional (2D) sodium dodecyl sulfate (SDS) polyacrylamide gel electrophoresis (PAGE) was first described in 1975 [15] and combined with mass spectrometry (MS) maintains its place as one of the most effective technologies for proteomic analysis [14,16,17].

**Figure 1:**
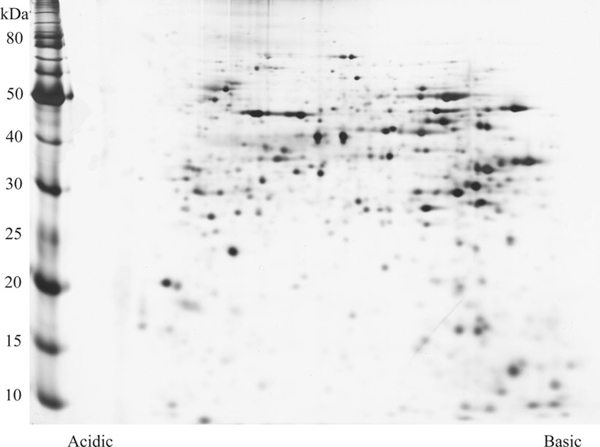
**Two-dimentional polyacrylamide gel (T—12%, C—3%; pH 3–10, non-linear gradient) of rabbit retinal tissue**. Proteins are separated according to both their charge and their molecular mass.

2D-PAGE separates proteins in two distinct steps. The protein mixture under study is initially applied to the first dimension gel strip that separates according to charge through isoelectric focussing (IEF). The strip is then applied to second dimension SDS-PAGE where the proteins are further separated according to their molecular masses (Figure 1). The relatively small amounts of sample needed for successful 2D-PAGE analysis is a particular advantage for ophthalmic research where tissue can be limited [4].

Conventional biochemical techniques often study individual proteins or isolated pathways; however, with 2D-PAGE, thousands of proteins can be separated from a single sample in a few steps. The proteins in the gels are then visualised with each resulting spot ideally representing a single protein. These can be studied with the aid of sophisticated computer analysis software to determine the spots of interest to be identified, usually with MS [14,15,17].

Proteins that have undergone PTM, for example phosphorylation or glycosylation, as well as the different forms arising from alternative splicing can be detected by 2D-PAGE. The 2D-PAGE technique is especially suited for resolving and visualising PTMs since many of these cause a difference in both the charge and mass of the protein [14,18]. These chemical and structural modifications may have an immense bearing on the protein's function, and are not apparent from genomic or transcriptomic studies [9,11,16,18].

Other methods of protein separation, involving electrophoresis, IEF and chromatography are available; however, here we mainly focus on the core technology of 2D-PAGE, summarising techniques specially optimised for the cells and tissues of the eye. Rather than a detailed laboratory protocol, the purpose of this article is to provide a methodological overview and useful reference to this scientific field.

## 2. General Sample Preparation

The preservation of biological state and sample quality prior to proteomic processing and analysis are extremely important. The proteins should be protected against loss or change as a consequence of proteolytic degradation. Ideally, ocular samples should be snap-frozen in liquid nitrogen immediately and stored at approximately -80°C until further use. In practise this is not always possible, especially in a clinical setting. Therefore, at the very least samples should be immediately placed on ice until deep freezing which should not be delayed beyond approximately 30 min [19,20]. The use of specific protease inhibitors may also be considered.

Cell disruption techniques are necessary when intracellular proteins are being analysed. Depending on the sample type, cell wall lysis can be induced by a variety of methods that include the use of freeze–thawing, hyperosmotic solutions, ultrasonication, detergents and enzymes. A sample may be fractionated prior to 2D-PAGE separation with for instance, centrifugation or chromatography. These techniques allow specific organelles or groups of proteins to be selected for optimised analysis, and allow less abundant proteins to be more clearly resolved when complex cells or tissue samples are being analysed such as those present in the different components of the eye. In addition, the use of narrow range immobilised pH gradient (IPG) strips (ultra-zoom gels) increases the number of proteins that can be resolved, conferring selection abilities in the first dimension [16,21,22].

Appropriate preparation of the sample is essential for 2D-PAGE to produce reliable results. Ocular tissues and cells require processing that should result in the complete solubilisation, disaggregation, reduction and denaturation of the proteins [23]. The sample buffer generally consists of concentrated urea and one or more detergents. Urea denatures the proteins for the first dimension, unfolding the proteins and exposing all the ionisable groups and prevents aggregation caused by hydrophobic interactions. Urea is ideal since it has no effect on the native charge of the proteins. Thiourea can be helpful if the proteins in a sample are difficult to solubilise. The denaturing effects of the sample solution itself is often able to prevent the action of many proteases, although as previously mentioned specific protease inhibitors may also be indicated [21-24].

The sample solution also includes carrier ampholines or IPG buffers that enhance protein solubilisation and can increase precipitation of unwanted nucleic acids during centrifugation. Other additions include reducing agents, commonly dithiothreitol (DTT) or β-mercaptoethanol (BME), which disrupt disulphide bridges to allow full denaturation of the proteins [21,24].

Membrane proteins have been shown to account for a significant proportion of human proteins and drug targets [25]. These proteins are involved in a wide range of important cellular processes that include signal transduction, transport (proteins, hormones, ions and metabolites) and cell division. For example, the visual pigment rhodopsin is a G-protein-coupled (transducin) receptor that comprises seven transmembrane helices. Mitochondrial membrane proteins such as cytochrome *c* oxidase are vital for many of the key functions of the mitochondrion that include oxidative phosphorylation, regulation of cellular metabolism and apoptosis. However, these inherently hydrophobic proteins can pose a particular challenge for 2D-PAGE as a result of their low solubility and tendency to aggregate, and many sample preparation protocols have been developed to specifically address this issue with varying success. The use of the cationic detergent 16-BAC in the first dimension followed by the anionic SDS detergent in the second dimension has been shown to improve 2D resolution of hydrophobic proteins [25-27].

## 3. Ocular Sample Preparation

In addition to the general sample preparation points discussed above, each of the individual parts of the eye require a more empirical approach. Although ideal ocular sample preparation methods for proteomic analysis are not presently clearly defined, here we offer an insight into practises that have worked well.

### 3.1. Cornea

The cornea can be processed as a whole or separately by each of its anatomical components (epithelium, basement membrane zone, stroma, Descemet's membrane and endothelium). Thompson et al. [28] investigated the proteomic changes of rat corneas following angiogenesis induced by silver nitrate-cautery. They harvested 60 rat eyes and dissected their corneoscleral rims. The tissue was solubilised in sample buffer by grinding with a liquid-nitrogen-chilled mortar and pestle, before centrifugation [28,29]. The sample buffer consisted of 9 M urea, 2% CHAPS, 2% DTT, 0.1% SDS, trace bromophenol blue in addition to a 0.5 tablet/ml of Complete Protease Inhibitor Cocktail (Roche, Basel, Switzerland). This protease inhibitor works against many proteases including cysteine-, serine- and metalloproteases. Following centrifugation to remove particulates, an IEF buffer (9 M urea, 2% CHAPS, 2% DTT, 1% Pharmalyte, pH 3–10) was added before loading onto IPG stips, pH 4–7. The 2D 10% polyacrylamide gels were silver stained without glutaraldehyde during fixation in order to prevent the problem of protein cross linkage which can impede MS-based identification. This optimised preparation for rat corneas was consistently able to produce high-quality 2D-gels for protein visualisation, analysis and subsequent mass spectrometric identification. Indeed, over 100 proteins were shown to change their expression in response to cauterisation.

This research group performed a study on potential markers of keratoconus [30]. Human corneal epithelial cells were isolated from patients shortly before undergoing corneal transplantations. The central 8 mm of the patient's cornea was coagulated with absolute ethanol and washed in PBS. The epithelium was then gently scraped off with a scalpel and quickly dissolved in 4 M guanidine thiocyanate (dissolved in 25 mM citrate, 0.5% lauroylsarcosine and 100 mM BME), followed by immediate snap-freezing in liquid nitrogen [30,31]. Two different sample preparation protocols were used; direct addition of lysis buffer (9 M urea, 2% Triton X-100, 0.13 M DTT and 2% IPG buffer), or pre-treatment with the ready-to-use reagent RNAzol B (Wak Chemie Medical, Steinbach, Germany). The pre-treated samples homogenised and lysed in the RNAzol B solution (phenol and guanidine thiocyanate) were separated into an aqueous and organic phase by the addition of chloroform. Subsequent centrifugation was then used to remove the protein from the aqueous containing RNA. The proteins were precipitated by the mixing of two volumes of ice-cold 99.9% ethanol. After incubation on ice for 1 h, samples were centrifuged at 14,000×*g* for 10 min. The supernatant containing RNA was discharged whilst the pellet was dissolved in the lysis buffer mentioned. The IEF buffer consisted of 8 M urea, 0.02 M CHAPS, 0.02 M DTT and 2% IPG buffer, using non-linear IPG strips, pH 3–10. Six percent and 12% polyacrylamide gels were utilised for the second dimension separation to accommodate a greater range of molecular masses with silver staining revealing hundreds of resolved proteins. It was readily apparent that there was far less smear and background staining on the gels loaded with the RNAzol-B-treated protein samples. To check for loss of proteins in the RNA-depleted samples, the chloroform phase and RNA-containing aqueous phase were run for 2D-PAGE analysis. Abundant proteins were also found in the aqueous buffer to some extent. In addition, on direct comparison of the 2D-gels with samples directly lysed in buffer and those pre-treated for RNA removal, there were a few proteins that were not represented in either group. Overall, RNA removal was effective in producing 2D-gels with less streaking which would help to resolve, analyse and identify certain proteins; however, this led to the unintentional removal of some others.

Aside from nucleic acids, there are other interfering substances that can cause problems with 2D-PAGE. For instance, high salt concentrations may affect protein solubility and impair electrical current during IEF. Jurkunas et al. [32] attempted to avoid this difficulty in their proteomic study of human corneal endothelial cells affected by Fuchs' endothelial dystrophy by subjecting the cells to an additional washing step with HEPES buffer. The primary protein buffer added to the samples consisted of 5 M urea, 2 M thiourea, 2% CHAPS, 2% SB3-10, 40 mM Tris, 0.2% ampholyte (pH 3–10) and 1 mM TBP. IEF was undertaken on IPG strips, pH 3–10. A preliminary study of pooled samples confirmed the reproducibility of their 2D protein separation, and the 8–16% precast polyacrylamide gels (Bio-Rad, Hercules, USA) stained with SYPRO ruby in the final study were of very high quality for subsequent image analysis. Although the extra washing step to remove salt was rather simple, it does not imply that it is less effective than other more sophisticated techniques such as protein precipitation, ultra filtration and dialysis, which have been shown to cause significant protein loss [22].

Karring et al. [33] conducted a proteome analysis of normal intact human corneas using a variety of protocols to optimise extraction and separation of proteins. For 2D-PAGE and MALDI-MS analysis, water-soluble proteins were extracted from corneal powder with 100 mM NaCl, 50 mM Tris–HCl, pH 7.4, containing a cocktail of protease inhibitors (2 mM EDTA, 2 mM 1, 10-phenanthroline, 40 μM E-64 and 2 mM AEBSF). However, the proteins found on the silver stained 12.5% 2D-gels were largely the same as those extracted using a more standard lysis buffer (5 M urea, 2 M thiourea, 2% CHAPS, 2% SB3-10, 10 mM DTT, 2 mM EDTA, 2 mM 1,10-phenanthroline, 40 μM E-64, 2 mM AEBSF, 0.5% IPG buffer of pH 3.5–5.0, 4–7 or 6–11 and bromophenol blue).

To facilitate the identification of insoluble proteins and those at the extremes of molecular mass and isoelectric point (pI), Karring and colleagues used CNBr to chemically digest the cornea followed by trypsin digestion to produce peptides suitable for liquid chromatography (LC)–electrospray ionisation (ESI)–MS/MS analysis. However, only around 30 proteins were identified using this method. The authors postulated that this was due to the large number of peptides derived from highly abundant proteins such as collagens. In an effort to overcome this problem proteins were fractionated by 1D-PAGE prior to tryptic digestion, which led to over 100 identifications with LC-ESI-MS/MS. However, they found that this method was not suitable for proteins at the extremes of molecular mass or proteins such as collagen that are not soluble in SDS sample buffer. All in all, 141 distinct proteins were identified with many that were never previously found in the mammalian cornea using direct identification methods [33].

### 3.2. Conjunctiva

Shimmura et al. [34] performed 2D-PAGE comparisons of the supernatants from cultured conjunctival, limbal and central corneal fibroblasts in a search for stem cell markers. Supernatants were placed in ultra filtration tubes and centrifuged so that proteins of less than 3 kDa were removed and those above concentrated. The lysis buffer consisted of 8 M urea, 2% Nonidet P-40, 2% ampholine (pH 3.5–10), 5% BME and 1 tablet/10 ml Complete Mini EDTA-free protease inhibitor (Roche, Basel, Switzerland). IEF was performed using a disc gel, pH 3.5–10 (Nihon Eido, Tokyo, Japan) followed by second dimension separation upon a 16.8% polyacrylamide gel. The proteins were then visualised with Coomassie brilliant blue staining. Since the supernatant was relatively low in protein levels compared to the pelleted cells, some of the 2D-gels could not be analysed despite condensing the samples. Precipitation techniques are an alternative method of protein concentration.

### 3.3. Sclera

Sclera is a fibrous connective tissue that can prove especially difficult for protein extraction. Patel and colleagues [35] studied a variety of protocols to optimise protein extraction primarily from porcine scleral tissue for 1D-PAGE (LC-ESI-MS/MS). The authors found that the recently developed technique of pressure cycling was able to recover more proteins from porcine scleral tissue than classical methods of homogenisation. Pressure cycling was performed for 60 cycles of 20 s at 35,000 psi followed by a sudden drop to atmospheric pressure, maintained for 5 s. Protein recovery was estimated using Bradford's method. Pressure cycling was able to extract more protein in the posterior sclera, compared to anterior or peripapillary sclera, suggesting regional variations in scleral protein composition. Furthermore, aqueous-organic phase partitioning was found to improve separation and resolution on SDS-PAGE. The authors also investigated the effects of a variety of ionic, non-ionic and zwitterionic detergents upon different regions of the porcine eye using both Bradford's and BCA protein assays. The combination of 0.5% Triton X-100, 0.5% Tween-20 and 0.1% Genapol C-100 was shown to increase protein extraction from the sclera.

Lu et al. [36] performed 2D-PAGE upon normal anterior and posterior human scleral fibroblasts. Cells were cultured, ultrasonicated and centrifuged. The resultant supernatant containing the solubilised protein was mixed in 8 M urea, 2% CHAPS, 20 mM DTT, 0.5% IPG buffer and bromophenol blue. IEF was undertaken with IPG strips, pH 3–10 and 12% polyacrylamide gels were utilised for the second dimension. The Coomassie-R250 stained gels were of high quality, resulting in 455 and 453 protein spots from anterior and posterior scleral fibroblast samples respectively, with 19 spots demonstrating a fivefold or more level of differential expression on analysis.

### 3.4. Trabecular Meshwork

Proteomic analysis of the trabecular meshwork (TM) has been primarily studied in an effort to further define the role of this structure in glaucoma pathophysiology [37-41]. Indeed, the TM is the main site of resistance to the outflow of aqueous humour from the eye.

Zhang and colleagues [37] undertook a 2D-PAGE comparison of the membrane extracts of normal and glaucomatous TM cells. Normal TM tissue was obtained from five human donor eyes within 24 h of death and isolated under microscopic dissection. TM from five primary open-angle glaucoma patients was acquired within 1 h following standard surgical trabeculectomy. TM cells were cultured in Dulbecco's modified Eagle's medium with 10% foetal bovine serum, 2 mM glutamine and antibiotics maintained at 37°C in 5% CO_2_. Membrane and cytoplasm TM cell extracts were obtained by centrifugation of the TM cells at 1,500×*g* for 5 min before resuspension in lysis buffer (50 mM tris/HCL, pH 7.4, 1% Triton X-100 and 1% protease inhibitor cocktail) followed by liquid nitrogen freezing and thawing at room temperature. A cytoplasmic supernatant was obtained by centrifugation at 200,000×*g* for 45 min. The pellets were washed in ice-cold lysis buffer and resuspended in ice-cold 100 mM Tris/HCL (pH 7.4). The supernatant was then centrifuged at 50,000×*g* for 20 min resulting in a membrane pellet, which was resuspended in 100 mM Tris/HCL (pH 7.4). Membrane samples (100 μg) were dissolved in 7 M urea, 2 M thiourea, 4% CHAPS, 20 mM Tris, 10 mM DTT, 0.2% ampholytes (pH 3–10) and a trace of bromchlorphenol blue before IEF, upon IPG strips, pH 3–10. Second dimension separation was undertaken on 13% polyacrylamide gels and was repeated in parallel at least three times for each sample. Following silver staining, 11 differentially expressed protein spots were found on the high quality 2D-gels.

### 3.5. Aqueous Humour

This research group performed a proteomic analysis of aqueous humour from patients with acute corneal graft rejection prior to any treatment [42]. Aqueous from cataract surgery patients with no other ocular conditions served as controls. IEF was performed with IPG strips pH 3–10. Preliminary trials indicated that 5 and 45 μL of aqueous humour were found to give the highest resolution of silver-stained protein spots in the 40–220 and 15–40 kDa molecular mass range, respectively. Therefore, both 6% and 12% polyacrylamide gels were used for second dimension separation. Samples were solubilised directly in a lysis buffer of 8.9 M urea, 2% Triton X-100, 2% IPG buffer and 0.13 M DTT. The IEF rehydration solution consisted of 8 M urea, 0.02 M CHAPS, 2% IPG buffer and 0.02 M DTT. Approximately 950 different protein spots were resolved across the 15–220 kDa range, with 31 spots found to be at least twofold differentially expressed.

Another study, performed by Duan et al. [43], undertook 2D-PAGE analysis upon aqueous humour of patients with high myopia using a protocol adapted from our studies [42]. Aqueous humour samples of 45 μL were solubilised in lysis buffer of 8 M urea, 4% CHAPS, 0.5% IPG buffer and rehydrated in 8 M urea, 2% CHAPS, 1% IPG buffer and 0.1 M DTT. First dimension IEF was performed with IPG strips pH 3–10 and second dimension separation was undertaken with 12% polyacrylamide gels. The final silver-stained 2D-gels proved to be of high quality with analysis revealing six protein spots at least twofold differentially expressed.

Grus and colleagues [44] analysed aqueous humour from patients with primary open-angle glaucoma and noted a similar protein profile to our study [42]. Their preliminary studies found the highest resolution of spots using 30 μL of aqueous humour with IPG strips pH 4–7 and 13.5% 2D polyacrylamide gels. Sample preparation was based on their previous 2D-PAGE studies of tears [45]. Aqueous humour was mixed with 8 M urea, 2% CHAPS, 0.28 DTT, 0.5% IPG buffer and 0.002% bromophenol blue before loading onto IPG strips. Approximately 225 proteins spots were resolved between a 10 and 80 kDa range on the silver stained gels, with results that were verifiable with protein chip arrays and ELISA.

### 3.6. Uvea

Uveal melanoma is the commonest primary intraocular malignant tumour in adults [46]. There is much interest in understanding the pathogenesis of this disease with a view to differentiating favourable and unfavourable tumours in terms of growth and metastasis [47]. Proteomic 2D-PAGE studies were recently applied to cell lines from primary uveal melanoma [48] and cell lines originating from the hepatic metastases [49]. In both studies the human uveal melanoma cells were similarly cultured in RPMI medium with 10% foetal bovine serum, 2 mM glutamine and antibiotics at 37°C in a 5% CO_2_ atmosphere. Pardo et al. [48] endeavoured to obtain a proteomic cell map of uveal melanoma. To disrupt the cells, sonication was performed in a lysis buffer of 5 M urea, 65 mM CHAPS, 0.15 M NDSB-256, 1 mM sodium vanadate, 0.1 mM sodium fluoride and 1 mM benzamidine. Following centrifugation the supernatant was removed, and 600 μg of protein was added in 375 μL of lysis buffer with IPG buffer. IEF was undertaken on four IPG strips, pH 3–10. The investigators chose to use a broad pI range 3–10 rather than zoom gels in an effort to obtain a global representation of the proteins. Approximately 1,150 protein spots were resolved in total on the 9–16% polyacrylamide gradient gels stained with the fluorescent dye OGT MP17, with around 85% reproducible across at least three of the four gels. Random excision and subsequent MS of 270 spots over the range of spot intensities yielded 683 distinct proteins.

This research group has recently performed 2D-PAGE LC-ESI-MS/MS analysis upon human uveal melanoma tissue with monosomy and disomy 3. Direct analysis of the tissue provides another window through which to view this tumour proteome [50].

### 3.7. Lens

The crystallins constitute more than 90% of total lenticular protein [51,52]. These water-soluble structural proteins undergo many PTMs throughout life, some of which may be required for their long-term functionality, whilst others can result in their aggregation and decreased solubility that may lead to lens opacification [52,53]. The 2D-PAGE method is able to allow detection and visualisation of proteins irrespective of PTM and may therefore be particularly useful for lens analysis [54]. Like the cornea, the lens can either be analysed in its entirety or dissected into its capsule, cortex and nucleus (and further subdivisions). Suitable sample preparation will also depend on the health and age of the lens under investigation [52].

Harrington et al. [55] divided lenticular matter into water soluble (WS) and insoluble (WI) portions, and further divided the WI into urea soluble (US) and insoluble (UI), prior to 2D-PAGE comparative study of normal and cataractous human lenses. A healthy lens and another with nuclear cataract were retrieved within 48 h following the death of a 68-year-old subject and 4–5 h following surgery in a 61-year-old subject, respectively. Lenses were initially stored in medium-199 without phenol red at -20°C. Each lens was decapsulated and suspended in a buffer of 50 mM Tris–HCl, pH 7.9, 1 mM DTT, 1 mM iodoacetamide (cysteine proteinase inhibitor) and 1 mM phenylmethylsulphonyl fluoride (PMSF; serine proteinase inhibitor). The investigators also used iodoacetamide for its ability to alkylate sulphydryl groups. The lens was homogenised in the sample solution using a tissue grinder before centrifugation at 15,000×*g* for 15 min. The supernatant was removed and the pellet rehomogenised and repeat centrifuged twice. The supernatants accounted for the WS protein fraction whilst the WI protein fraction was retained in the pellet. The WI fraction was suspended in 50 mM Tris–HCl, pH 7.9, 6 M urea and 5 mM DTT, and further homogenised and centrifuged. The resulting supernatants accounted for the water-insoluble–urea-soluble protein fraction (WI-US), and the pellet as the water-insoluble–urea-insoluble (WI-UI) protein fraction. Aliquots of 500–800 μg of WI-US and WI-UI lens protein were resolubilised in 5 M urea, 2 M thiourea, 2% CHAPS, 2% SB3-10, 2 mM TBP, 40 mM Tris, pH 8.0 before loading onto IPG strips, pH 3–10. The second dimension was undertaken upon 15% polyacrylamide gels, which were subsequently stained with Coomassie blue. Analysis revealed significant protein differences between the healthy and cataractous lenses, and between the WI-US and WI-UI proteins.

In another similar study Posner and colleagues [56] utilised 2D-PAGE to characterise the PTMs of the crystallins and obtain a proteome map in the zebrafish lens. Zebrafish adult lenses were homogenised in 8 M urea, 2% CHAPS, 50 mM DTT, 0.2% Bio-Lyte ampholyte pH 3–10 and 0.001% bromophenol blue. Following centrifugation at 15,000×*g* for 20 min, 150 μg of lens homogenate was focused on IPG strips at pH 3–10, 5–8 and 4–7. Another set of lenses were homogenised in 20 mM sodium phosphate also containing a protease inhibitor (Roche, Basel, Switzerland) and centrifuged to produce a soluble supernatant protein fraction and insoluble pellet fraction. Using the Bradford method 150 μg of each protein fraction was diluted in the above stated buffer and applied to IPG strips, pH 5–8. In all cases second dimension separation was undertaken on 12% polyacrylamide gels, which were subsequently Coomassie stained. Over 80 protein spots were resolved on the high-quality gels, the majority being the crystallins and their PTMs. Furthermore, certain crystallins especially truncated versions were found to be more abundant in the insoluble fraction.

### 3.8. Vitreous Body

There have been a number of vitreous 2D-PAGE studies for conditions such as uveitis [57,58] and diabetic retinopathy [59-62]. An important issue to consider with vitreous in particular is the problem of highly abundant proteins such as albumin and immunoglobulin possibly preventing the detection of lower abundant proteins [63,64]. Kim and co-workers [62] attempted to deplete certain proteins known to be highly abundant in biological fluids such as human plasma from patient vitreous samples as part of their proteomic study of proliferative diabetic retinopathy (PDR) involving 2D-PAGE. This was achieved with the immunoaffinity subtraction system, ProteomeLab IgY-12 spin column (Beckman Coulter, Fullerton, CA, USA) which aims to only remove: albumin, IgG, transferrin, fibrinogen, IgA, α2-macroglobulin, IgM, α1-antitrypsin, haptoglobin, orosomucoid, apolipoprotein A-I and apolipoprotein A-II from the sample. However, Liu and co-workers [65] evaluated this system and found that other proteins were also depleted from the sample, though when this occurred it was reproducible.

The low abundant proteins are obtained in the flow-through fraction whilst the 12 high abundant proteins are resin antibody bound within the column. The bound proteins were retrieved with the use of stripping (1 M glycine–HCl, pH 2.5) and neutralisation buffers (1 M Tris–HCl, pH 8.0). Both protein fractions were subsequently dialysed with the Slide-A-Lyzer (Pierce, Rockford, IL, USA) in an effort to remove salt and small molecules less than 3.5 kDa. The desalted protein samples were precipitated with TCA in acetone and centrifuged. The supernatant was discarded and the dried pellet rehydrated in 7 M urea, 2 M thiourea, 2% CHAPS, 60 mM DTT and IPG buffer, pH 3–10. IEF was undertaken on IPG strips, pH 4–7. Second dimension separation was undertaken on 10% polyacrylamide gels that were subsequently silver stained.

The low abundant protein fraction yielded 47 2D-gel spots for analysis, with only six spots able to be identified by MALDI-MS. Three of the six spots were identified as albumin. The authors explained that this very low protein spot identification might either be due to low protein spot concentration or low yields of low abundant protein. The high abundant protein gels resolved 116 spots with 87 successfully identified, most as expected being albumin in addition to transferrin, haptoglobin, apolipoprotein and α1-antitrypsin.

2D-PAGE analysis was also conducted on non-immunoaffinity-subtracted PDR vitreous. This yielded 69 spots for excision of which 54 spots were identified as 28 different proteins.

In effort to increase the number and diversity of protein identifications Kim and colleagues [62] employed 1D-fractionation and tandem MS using two ionisation methods. In this case only albumin and IgG were depleted from the vitreous using a ProteoExtract albumin/IgG removal kit (Calbiochem, San Diego, USA). With LC-MALDI-MS/MS fifty-four proteins were identified in the albumin/IgG depleted PDR vitreous and 49 proteins in the non-depleted PDR vitreous. However, LC-ESI-MS/MS identified 356 proteins in albumin/IgG depleted PDR vitreous and 136 proteins in the non-depleted PDR vitreous.

### 3.9. Neurosensory Retina

There is great interest in the protein changes of the neurosensory retina affected by diabetic retinopathy [66-68] and age-related macular degeneration (AMD) [69] the leading causes of blindness in the working age and elderly population, respectively. Ethen et al. [69] utilised 2D-PAGE to compare the proteome of central and peripheral neurosensory retina at different stages of AMD. Post-mortem eyes were snap-frozen in liquid nitrogen and stored at -80°C. The vitreous was removed whilst the eyes were still frozen to minimise contamination of the neurosensory retina. An 8-mm trephine punch was used to separate the macula from the peripheral retina. The neurosensory retina was then carefully peeled from the retinal pigment epithelium (RPE) and rinsed in PBS to minimise RPE cell contamination. Samples were homogenised in a medium containing 20% sucrose, 20 mM Tris–acetate (pH 7.2), 2 mM MgCl_2_, 10 mM glucose and 2% CHAPS. The homogenate was centrifuged and the supernatant retained. The resultant pellet was rehomogenised, centrifuged and the supernatant removed. Both supernatants were combined and 150 μg of retinal protein was processed for 2D-PAGE analysis. IEF was performed with IPG strips, pH 5–8 that were equilibrated for 10 min in 6 M urea, 2% SDS, 375 mM Tris–HCL (pH 8.8), 20% glycerol and 130 mM DTT, followed by 10 min in the same buffer with 135 mM iodoacetamide substituted for DTT. Analysis of the 12% polyacrylamide silver-stained gels revealed 584 and 524 well-resolved protein spots in the macula and peripheral neurosensory retina respectively, with 26 candidate proteins identified as being possibly related to the onset and progression of AMD.

This research group has performed 2D-PAGE on the neurosensory retina from a patient afflicted with Leber's congenital amaurosis (LCA) [70]. Immediately after enucleation the eye was divided along the horizontal meridian and a 4 × 4-mm section of neurosensory retina was carefully excised from the underlying RPE. The retinal anatomical location and area were matched to that of the seven normal controls to ensure standardised samples for optimal comparison. Samples were added to a lysis buffer consisting of 9 M urea, 2% DTT, 2% Triton X-100 and 2% IPG buffer. The IPG strips, pH 3–10 were rehydrated in 400 μL of buffer containing approximately 100 μg of protein in 8 M urea, 2% CHAPS, 0.3% DTT and 2% IPG buffer. Second dimension separation was undertaken on 15% polyacrylamide gels that were subsequently silver stained using a glutaraldehyde-free fixation method. Four differentially regulated proteins were identified as being possibly associated with LCA pathogenesis. Scleral tissue was used as an additional control since it is not thought to be involved in the pathology of LCA. Indeed, there was no significant difference in the 2D protein expression between the scleral tissue obtained from the LCA and normal eye.

### 3.10. Retinal Pigment Epithelium

There have been a number of proteomic studies of the RPE [71-76] and the extracellular deposits that can accumulate beneath this cellular layer termed drusen, which are associated with the development of AMD [77,78]. Using 2D-PAGE Nordgaard et al. investigated the global [71] and mitochondrial proteome [72] of RPE cells at different stages of AMD, complimenting the groups retinal studies [69]. After removal of the neurosensory retina, calcium- and magnesium-free phosphate-buffered saline was used to moisten the RPE cells to assist dislodgement from the underlying choroid. The cells were centrifuged at 1,100×*g* for 30 min and fractionated in 50 mM Tris (pH 7.8) and 2% CHAPS by freeze–thaw cycles and mechanical homogenisation. The lysate was centrifuged for 600×*g* for 15 min to pellet cell debris. Acetone precipitation was used to remove lipids and nucleic acids before the samples were resuspended in 8 M urea and 0.5% CHAPS. The protein concentration was determined and 140 μg of RPE protein was separated in the first dimension upon IPG strips, pH 5–8. Second dimension electrophoresis was undertaken with 12% polyacrylamide gels as described previously [69]. Approximately 440 protein spots were clearly resolved and analysed on the silver-stained gels [71].

The degree of RPE protein contamination with retinal photoreceptor outer segment (RPOS) was determined by quantifying the RPOS protein that was copurified with the RPE. The RPE cells were harvested, pelleted and resuspended in PBS as described above. The sample was subsequently layered over a 25% to 60% continuous sucrose gradient before centrifugation at 75,000×*g* for 75 min. The RPOS fraction was then removed from the gradient and brought up in 10–15 mL of 10 mM Tris and further centrifuged at 17,369×*g* for 30 min. The total RPOS protein amount was calculated as a percentage of the total protein yield from the corresponding sample, which on average was only around 5%. In addition, analysis revealed no linearity between AMD stage and degree of RPOS contamination suggesting that the contamination did not vary between the different AMD grades [71].

Nordgaard and colleagues [72] undertook a further 2D-PAGE analysis of the subproteome of enriched RPE mitochondria associated with different stages of AMD. RPE cells were homogenised in 0.5% Nonidet P-40, 1 mM PMSF, 20 mM HEPES (pH 7.5), 10 mM KCl, 1.5 mM MgCl_2_, 250 mM sucrose 1 mM EDTA and 1 mM EGTA. The homogenised tissue was freeze–thawed twice and the tissue suspension passed gently through a 26-gauge needle six times. Centrifugation at 600×*g* for 15 min cleared the lysate of nuclei and intact cells. The supernatant was retrieved, pellet rehomogenised and centrifuged. The resultant supernatant was combined with the first. Mitochondria were pelleted from the lysate by centrifugation at 13,000×*g* for 15 min. The pellet was then resuspended in 8 M urea, 2% CHAPS and 0.5% ASB-14. In an effort to increase the solubility of the membrane bound proteins, the mitochondrial fraction underwent two freeze–thaw cycles and incubation in a water bath sonicator for 30 min. Antibody probing for GFAP and CD34 antigens suggested little or no contamination of the mitochondrial samples with retina or choroid, respectively. 2D-PAGE was undertaken with 100 μg of mitochondrial protein loaded onto IPG strips, pH 3–11. Second dimension separation was performed with 12% polyacrylamide gels that were subsequently silver stained for protein spot visualisation. Approximately 440 protein spots were resolved in each gel, and the 222 spots that were consistently resolved were further analysed revealing eight differentially expressed spots.

## 4. Methods of Protein Visualisation

### 4.1. Protein Staining

To visualise the proteins in the gels, they must be stained or labelled. This is a vital step of the 2D-PAGE procedure with regard to the number, quantitation and subsequent comparison of proteins spots [79]. Indeed, it is the detection of resolved proteins rather than the degree of protein resolution that is a common limitation with proteomic analysis by 2D-PAGE [80]. A protein stain should bind irrespective of the protein's composition, relative hydrophobicity or ability to bind SDS, and work reproducibly over a broad range of protein concentrations without interfering with them so that subsequent analysis such as Edman sequencing or MS identification is not compromised [81]. In this section, we give a summary of the commonly used colourimetric and fluorescent dyes for ocular protein staining.

The most widely used dyes are Coomassie Brilliant Blue and silver nitrate. The sensitivity (100 ng) and specificity (high-background staining) of Coomassie may remain a problem despite the improved colloidal version; however, it is cost-effective, relatively simple to use and offers good compatibility with MS [11,80-84]. In addition, the application of infrared fluorescent detection rather than standard methods has been shown to significantly increase the sensitivity obtainable from colloidal Coomassie staining [80,85].

Silver nitrate offers excellent sensitivity (1–10 ng) and ease of use at a relatively low cost; however, the narrow linear dynamic range of silver, saturation of high abundance proteins and also negative staining of strong spots, can make relative quantitation difficult at times [11]. Additionally, due to common use of glutaraldehyde fixation for instance, which can cause covalent modification of proteins, in-gel digestion and peptide recovery necessary for MS-based protein identification can be impeded [86-88]. Indeed, there are over 150 published silver-staining protocols which aim to solve these problems whose success also depends upon which MS ionisation method is subsequently used [22,89-91]. Ammoniacal silver nitrate staining may also be a viable choice in certain situations [92,93] as well as other recently developed modifications [94]. A technique of note is the modified colloidal Coomassie Blue stain also known as blue silver, since it is reported to offer a sensitivity approaching that of silver with good compatibility with MS [95].

There are several fluorescent stains in use and most are simple to employ. Of these the SYPRO Ruby stain is considered one of the most effective and commonly used [80]. SYPRO Ruby has a sensitivity similar to silver nitrate, with a broad linear dynamic range (three orders of magnitude) and very good compatibility with in-gel digests for MS [86,88]. However, protein detection with SYPRO Ruby has been shown to have some degree of variance from protein to protein [96]. In addition, SYPRO Ruby is expensive and stained spots are invisible with the naked eye unless viewed under an ultraviolet or blue light source, making manual spot excision more hazardous and cumbersome [97]. Quinn and co-workers developed a 2D-PAGE proteome profile of normal and diabetic rat retina. After SYPRO Ruby staining and imaging, gels were again stained with Coomassie Blue aiding manual removal of protein spots from the gels. However, since Coomassie is less sensitive fewer spots were subsequently stained [66].

Deep Purple is a fluorescence-based stain which has been reported to be more sensitive than Sypro Ruby although background fluorescence and photoinstability are limiting factors [80,98,99].

### 4.2. Protein Labelling for 2D-DIGE

**Figure 2:**
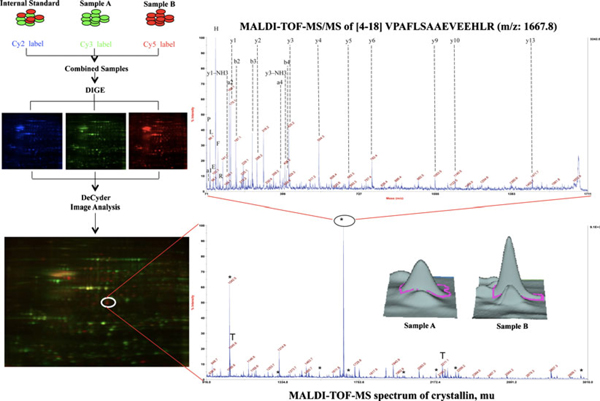
**Typical 2D-DIGE workflow for comparative expression proteomics**. The samples are labelled by fluorescent dyes (Cy 2, 3 and 5) and combined together prior to the IEF. The scanned fluorescent images are analysed by DeCyder™ analysis software using the normalisation by Cy2-labelled pool sample. The protein spots (e.g., crystallin mu) with statistical significance that express different expression profiles between the two groups are further analysed by MALDI-TOF-MS. The peptide mass fingerprint of crystallin (mu) shows the peptides marked with '*' matched to MASCOT search against the NCBInr primate database. The *x*- and *y*-axes show the mass to changed (*m*/*z*) ratio and the % abundance of the tryptic peptide fragments, respectively. Also depicted in the *inset* for crystallin (mu) mass spectrum is the representative 3-D protein-expression profiles for the two groups. Typically, the most-abuntant peptide fragment is sequenced by, additive series of the *y*- and *b*-ions generated as well as the immonium ions, MALDI-TOF-TOF (e.g., peptide fragment with *m*/*z* of 1,667.8) to confirm the identification of protein and charactirise the sites of PTM. Reprinted from Tannu and Hemby [22] with permission of Elsevier.

First described in 1997 [100], 2D fluorescence difference gel electrophoresis (DIGE) is a version of 2D-PAGE where the proteins in the sample are labelled prior to electrophoresis so avoiding the need for staining [101-105]. DIGE chemistry relies on the tagging of lysine and cysteine with minimal and saturation fluorescent cyanine dyes respectively (Figure 2). The minimal labelling strategy labels only around 5% of lysine residues thus limiting any decrease in protein solubility, apparent increase in molecular mass [105,106] and interference with subsequent tryptic cleavage (discussed later). 2D-DIGE can offer sensitivity up to 0.5 fmol of protein, a broad linear dynamic range of four orders of magnitude, and labelling that does not interfere with subsequent identification by MS [101,102]. One of the great advantages of this system is that up to three samples can be run on the same gel simultaneously, thereby reducing the number of gels needed. The different proteins can then be visualised separately by utilising the specific excitation and emission wavelengths of the dyes. An internal standard, made from a pooling of all the samples in the experiment, comprises one of the three samples on the gel. The internal standard is labelled with Cy 2 for example and run together with individual experimental and control samples which are labelled with the other dyes (e.g. Cy 3 or Cy 5). Therefore, every protein from each of the samples is represented on the internal standard to which it can be compared and relative quantitation calculated. Since the internal standard is normalised across all the gels, separation of inter-gel experimental variation from biological variation is possible [11,102].

In all, 2D-DIGE offers a more reliable and reproducible method of relative protein quantitation [107]. Proteins with a higher abundance of lysine residues will be (in the case of the minimal procedure) labelled more heavily than proteins with fewer lysine groups irrespective of true protein amount. However, since protein spot intensity is related to the intensity of the corresponding spot in the internal standard this will not pose a problem with regard to relative protein quantitation [103,107]. A potential drawback which follows with the dependence upon a specific amino acid for tagging is that in the absence of lysine no labelling will occur [11]. Another important consideration is that labelled proteins are approximately 0.5 kDa higher in molecular mass leading to slightly different positions on the 2D-gels compared to the unlabelled [11]. Since only a small percentage of proteins are labelled it should be the unlabelled protein that is excised for mass spectrometric analysis. To ensure this, further staining of the gel following 2D-electrophoresis with another agent such as silver nitrate or SYPRO Ruby is necessary for spot picking and cutting, especially for lower molecular mass proteins [11,106]. Protein labelling of cysteine residues at saturating conditions in the first instance may also be considered to avoid this extra staining step. However, since the dyes react with cysteine rather than lysine residues the gel image can appear quite different due to the differing amounts of these amino acids. In addition, the approximately 13% of eukaryotic proteins that do not contain cysteine residues will not be labelled [11]. Furthermore, the DIGE system (GE Healthcare) requires specialised reagents, scanners and software that may not be affordable for many laboratories; however, it is an effective proteomic tool that continues to be applied in ophthalmic research [38,68,108-111].

Fort et al. [68] used 2D-DIGE and iTRAQ (covered later) proteomic techniques as well as immunological and genetic experiments to characterise the protein changes in the retina of Sprague–Dawley diabetic rats receiving no insulin and those treated with a combination of different insulin regimens compared with controls over time. Diabetes was induced in this rat model by intraperitoneal injection with streptozotocin. During sample preparation, lipids and nucleic acids were removed from the retinal samples by precipitation using The 2D-Cleanup Kit (GE Healthcare). Samples underwent IEF (pH 3–10) and second dimension separation upon 10% polyacrylamide gels. SYPRO Ruby staining was used for spot picking following electrophoresis. Analysis of the 2D-DIGE experiments upon the 8-week diabetic rat retinas compared with age-matched controls with or without treatment revealed over 30 differentially expressed, mostly decreased, protein spots. The nine spots that were increased in expression were located in the same region upon the 2D-gel and identified as different crystalline proteins or isoforms by mass spectrometry (MALDI-MS/MS). The increased crystalline expression was confirmed with iTRAQ analysis and further characterised with immunoblotting, immunohistochemistry and real-time RT-PCR related to the time course and treatment of the induced diabetes.

García-Ramírez and colleagues [108] applied 2D-DIGE to human vitreous affected with PDR. Affinity chromatography was used to remove albumin and IgG (GE Healthcare) from the vitreous samples that were further purified by acetone precipitation. Approximately 1,400 protein spots were resolved of which 41 were found to be greater than 1.4-fold differentially expressed.

## 5. 2D-Page Image Acquisition and Analysis

Prior to post-experimental quantitative analysis, a digitised image of the gel is required. There is a range of devices available with the most commonly used being a densitometer, camera system, phosphor imager or fluorescence scanner.

Detailed evaluation of the scanned images is made possible by a variety of computer image analysis software packages. The sophisticated algorithms allow the comparative analysis and storage of the complex 2D-gel data. Some of the most commonly used programmes at the present time include PDQuest (Bio-Rad, USA), Image Master 2D Platinum (GE Healthcare), Delta2D (DECODON, Germany) and Progenesis SameSpots (Nonlinear Dynamics, USA). The latter is compatible with both 2D-PAGE and 2D-DIGE. Each programme has its advantages and disadvantages, and direct comparisons between them can be difficult [96,112-115].

The general working principles of analysis in a classical 2D-PAGE approach involves the comparison of thousands of proteins between different groups to find those that are differentially expressed. Typically, the first step is noise reduction, by correcting the raw image data and accurately subtracting the gel background. Spots are then detected, volumes normalised to correct for experimental variances, followed by inter-gel matching and relative quantitation. A reference or master gel is chosen to which all other gels are compared. If the matching of spots is inaccurate conclusions about their differential expression cannot be drawn. To fulfil the gold standard of analysis, manual editing and verification of the matched spots is generally required, which can be very time consuming. With this in mind the Progenesis software programmes were designed to offer superior automation of spot detection and matching. Indeed, Progenesis Workstation has shown comparable results to PDQuest's more manual approach, but only when spots are well resolved [114]. A standardised evaluation test of the various analysis programmes has yet to be developed [105].

## 6. Protein Identification and Verification

After establishing the differential expression of the protein spots they can be excised from the gel and processed for identification. Gel excision can be performed manually or by way of an automated cutter. Some of the analysis software tools, for example PDQuest, can be integrated with an automated gel cutter to increase throughput and accuracy. The excised protein spots can be characterised by a number of means such as Edman sequencing and amino acid composition [88,116]; however, MS is the method of choice [11,117].

There have been many great advances in MS over the years, consolidating its position as the leading technology in the identification and characterisation of proteins within biological systems [117-120]. Indeed, MS is a highly sensitive and rapid technique for the identification of proteins that have previously undergone separation commonly with 2D-PAGE. The proteins in the excised gel pieces are usually digested to generate peptides for MS analysis, since extraction of whole proteins from gels is inefficient [88]. The peptides recovered should be purified, since the detergents, salts and buffer contaminants can interfere with MS. These substances can be removed by various procedures including liquid chromatography [14,88]. Trypsin, typically used in protein identification, is a serine protease that cleaves proteins at the C terminus of lysine and arginine residues except when followed by proline, thereby generating a fingerprint of peptide masses with an average size of 800–2,000 Da that can be searched in databases or further sequenced by tandem MS [21]. This combination of mass spectrometric identification of digested samples, with subsequent comparisons with established databases is sometimes referred to as 'bottom-up' MS. In comparison, the novel and exciting 'top-down' approach involves the fragmentation of intact (non-digested) proteins within the mass spectrometer, although this technique is not sufficiently developed at present [119,121].

**Figure 3:**
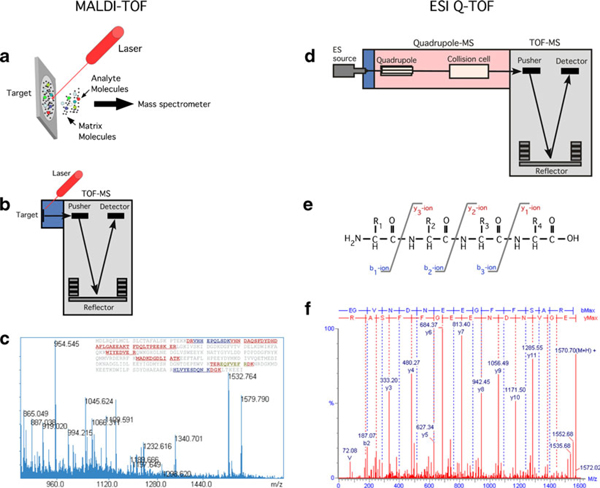
**Principles of mass spectrometry**. **a** MALDI-TOF mass spectrometer. The sample is co-crystallised with matrix molecules as a dry sample on the plate. The peptides are brought to an ionised gas phase by a laser pulse. **b** The ionised peptides are analysed in the time-of-flight (TOF) unit in the mass spectrometer giving a peptide mass fingerprint. **c** If the sample is pure enough, the peptide mass fingerprint can be used to search DNA and protein databases for identification. **d** Tandem mass spectrometry (MS/MS) as obtained by a Q-TOF mass spectrometer. The sample is ionised at atmospheric pressure by electrospray ionisation (ES source). The ions enter the vacuum system through the sampling cone and, in the quadrupole section ions, of a particular *m*/*z* are selected and fragmented in the collision cell. **e** In the collision cell, peptides are mainly fragmented at the peptide bonds producing b type (*blue*) and y type (*red*) ions. The masses of the resulting peptide fragments are measured in the TOF unit. **f** Example of a collision-induced spectrum with the amino acid sequence given as detected from the N-terminal (b type ions) and from the C-terminal (y type ions). Reprinted from Honoré et al. [11] with permission of John Wiley & Sons, Inc. The mass fingerprint (*C*) is reprinted from Honoré [134] with permission of Expert Reviews Ltd.

There are several combinations of MS instrumentation available, each with its own set of advantages and limitations. Generally, a mass spectrometer consists of an ion source, a mass analyser and a detection system. The two main ways of volatising and ionising proteins or peptides are matrix-assisted laser desorption ionisation (MALDI) and electrospray ionisation (ESI). With MALDI, the peptides/proteins are co-crystallised with a dry matrix on a plate. The molecules are brought into an ionised gaseous phase by way of a laser, which are subsequently analysed in the MS. The more complex ESI analyses molecules in solution and it can therefore be easily combined with liquid chromatography, thus providing an additional separation technique prior to mass spectrometric analysis [11]. A range of mass analysers is commonly used: time-of-flight (TOF), quadrupole, ion trap, high-resolution orbitrap and Fourier transform ion cyclotron resonance. Each one works differently, having their own strengths and weaknesses and can be used alone or in combination [11,117] (Figure 3).

Results from a proteomic experiment should not stand alone. At least one confirmatory test is necessary, commonly western blotting, immunohistochemistry or ELISA which all rely on antibody–antigen reactions. Western blotting aims to detect a specific protein in a sample that has been separated according to molecular mass, and provides semi-quantitative validation of the expression levels. Though the protein molecular mass is not apparent with immunohistochemistry, this technique is able to show the localisation of the protein within the cell and tissue architecture. ELISA offers a more quantitative measurement of the protein within the sample. However, the common difficulty encountered with these immunological methods is commercially acquiring or self-producing specific antibodies with a high affinity that work well in the particular system used.

## 7. Alternative Approaches in Ocular Proteomics

Non-gel-based MS offers an alternative way of studying ocular proteomics [22]. These include multi-dimensional protein identification technology (MuDPIT) [122], isotope-coded affinity tags (ICAT) [123,124], isobaric tags for relative and absolute quantitation (iTRAQ) [125-127] and stable isotope labelling with amino acids in cell culture (SILAC) [12,128,129].

The MuDPIT method capitalises on a protein's/peptide's unique physical characteristics of charge and hydrophobicity by utilising a LC system usually composed of a strong cation exchange and reverse phase chromatography column that is coupled with MS and database searching. The system works upon the total raw protein content of the sample that has usually been tryptically digested. This has allowed more proteins at the extremes of pI and molecular mass, as well as membrane proteins to be identified, all of which may be difficult for 2D-PAGE [122].

**Figure 4:**
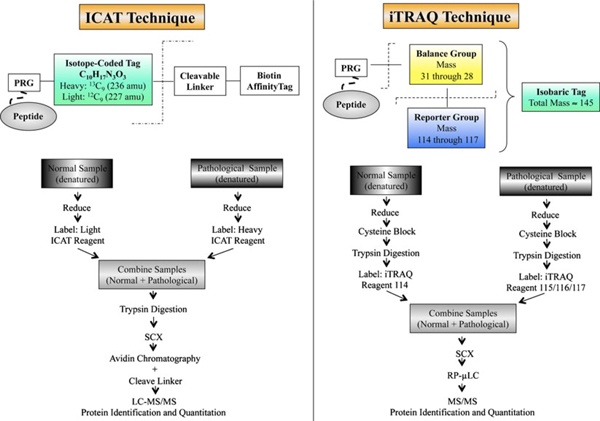
**Non-gel-based approach for analysis of expression proteomes**. The most commonly used, commercially available, quantitation methods coupled with the MDLC approaches of comparative proteomics are depicted. The figure shows the ICAT and iTRAQ reagent structures. The peptide-reactive group (PRG) covalently links iTRAQ as well as the ICAT reagent isobaric tag with the lysine side chain and N-terminal group of peptide. The fragmentation shown with *dashed lines* occurs during MS/MS. The balance group of the iTRAQ reagent undergoes neutral loss, and the resultant reporter group (114–117) peaks in the low-mass region are used for relative quantitation. Reprinted from Tannu and Hemby [22] with permission of Elsevier.

However, relative quantitation of protein expression between samples can be difficult with the MuDPIT technique, something that ICAT, iTRAQ and SILAC have attempted to address. ICAT labels cysteine residues of proteins with one of two mass tags differing by, e.g. 9 Da, whilst iTRAQ labels the N terminus of all peptides, and lysine residues with tags of identical mass with differing design [123,125] (Figure 4). With SILAC, labelling of proteins takes place during cell culture where one population of cells are grown in a medium containing a 'light' amino acid and another in a medium with a 'heavy' amino acid [128]. This type of chemical labelling of proteins (ICAT) and peptides (iTRAQ) and metabolic labelling of proteins in cultured cells (SILAC) results in differential ion intensities that can be detected by MS for relative quantitation.

Allison and colleagues [124] undertook a study of the effects of thyroid hormone on retinal development in a rainbow trout model. The authors were particularly interested in opsins, which can act as markers of photoreceptor differentiation. However, they found that these membrane bound proteins might not be able to be reliably detected using 2D-PAGE. They therefore opted to study the fish retina using ICAT and LC-ESI-MS/MS. With this method, 1,684 unique peptides including those of opsins were detected. Indeed, significant expression changes were found with four of the five opsin classes of the rainbow trout retina when comparing thyroid hormone-treated and untreated fish.

In another study, the levels of the anti-microbial protein defensin (NP-2) were measured in a rabbit model of corneal injury using iTRAQ with LC-ESI-MS/MS [126]. Tears were collected from the rabbits prior to injury and after corneal wounding days 1–3. Each of the four 5-μL samples was labelled with different iTRAQ reagents before being combined for analysis. As a further control iTRAQ was also used to measure expression levels of lipophilin AL, an abundant tear protein that has been shown in previous studies to remain relatively constant before and after corneal wounding. The levels of NP-2 increased after corneal wounding and decreased as healing progressed, whilst lipophilin AL remained stable throughout.

An et al. [129] used SILAC and LC-ESI-MS/MS to examine the differential protein secretion of RPE cells in relation to drusen and pathological changes of AMD. The authors obtained healthy age-matched donor RPE cells with the wild-type complement factor H (CFH) genotype, and AMD donor RPE cells with the homozygous and heterozygous CFH genotype for culture in isotopically unlabelled and heavy amino acids respectively (^13^C_6_-Arg and ^13^C_6_, ^15^N_2_-Lys). This procedure identified many differentially secreted proteins involved in tissue development, angiogenesis, complement regulation and protein aggregation.

Choe et al. [130] compared the efficacy of 2D-PAGE using SYPRO Ruby staining, with iTRAQ and concluded that both techniques had similar consistency in measurements (CV <31% 2D-PAGE vs. <24% iTRAQ). Similarly, a recent study on human epithelial ovarian cancer found that the combination of 2D-PAGE with SYPRO Ruby staining and iTRAQ produced complimentary results [131].

A comparative study of 2D-DIGE, ICAT and iTRAQ showed that all three techniques produced fairly accurate quantitative results [132]. The global tagging of iTRAQ offered better sensitivity than the limited cysteine tagging of ICAT since more peptides were detected for a given protein. Comigration of multiple proteins into a single 2D-gel spot posed problems for spot boundary and relative quantitation calculations with the DIGE technique. However, DIGE was found to have comparable sensitivity with ICAT [132]. DIGE incorporates a dynamic range of four orders of magnitude where as in most cases MS is below three orders of magnitude [101,102]. In addition, just as comigration of proteins upon the 2D-gel can cause quantitation problems, a similar difficulty exists with MS-based quantitation due to overlapping signals from different ion species unless high-resolution MS is used [102,133]. It is also important to note that the relative quantitations made with 2D-DIGE are at the protein level, in contrast to MS where it is based on peptides. Furthermore, the use of proteolysis to generate lower molecular mass peptides can lose essential information necessary to distinguish the different protein isoforms arising from alternative splicing, PTM and proteolytic cleavage.

## 8. Closing Remarks

The use of appropriate sample preparation is essential to ensure the results obtained from a proteomic experiment are sound. The investigator must be clear on which type of ocular sample and proteins are under scrutiny and apply the necessary preparatory steps. However, it must be kept in mind that the addition of further sample preparation steps may improve the perceived quality of the results in one way by reducing complexity, but possibly bias the outcome by causing inadvertent, and perhaps irreproducible, loss of certain proteins.

Despite the technological advances, presently no strategy used alone or in combination can detect, quantify and identify the enormous protein profile present even in a single cell type. However, the current mass spectrometric-based proteomic techniques coupled with or without gel electrophoresis, especially with targeted approaches, hold the promise to further elucidate ocular disease processes and lead to the discovery of strong novel biomarkers and therapeutic targets useful in many ophthalmic conditions.

## Abbreviations

1D: One-dimensional; BME: ß-mercaptoethanol; 2D: Two-dimensional; 16-BAC: Benzyldimethyl-*n*-hexadecylammonium chloride; AEBSF: 4-(2-Aminoethyl) benzenesulphonyl fluoride hydrochloride; AMD: Age-related macular degeneration; ASB-14: Amidosulphobetaine-14; BCA: Bicinchoninic acid; CD: Cluster of differentiation molecule; CHAPS: 3-[(3-Cholamidopropyl)dimethylammonio]-1-propanesulphonate; CNBr: Cyanogen bromide; CV: Coefficient of variation; DIGE: Difference gel electrophoresis *alternatively* difference in gel electrophoresis; DTT: Dithiothreitol; E-64: Trans-epoxysuccinyl-L-leucylamido(4-guanidino)butane; EDTA: Ethylene diamine tetraacetic acid; EGTA: Ethylene glycol tetraacetic acid; ELISA: Enzyme-linked immunosorbent assay; ESI: Electrospray ionisation; FTICR: Fourier transform ion cyclotron resonance; GFAP: Glial fibrillary acidic protein; HEPES: 4-(2-Hydroxyethyl)-1-piperazineethanesulfonic acid; ICAT: Isotope-coded affinity tags; IEF: Isoelectric focusing; IPG: Immobilised pH gradient; IT: Ion trap; iTRAQ: Isobaric tags for relative and absolute quantitation; kDa: Kilodalton; LC: Liquid chromatography; MALDI: Matrix-assisted laser desorption ionisation; MDLC: Multi-dimensional liquid chromatography; MS: Mass spectrometry; MuDPIT: Multi-dimensional protein identification technology; NDSB-256: Dimethylbenzylammonium propane sulphonate; PAGE: Polyacrylamide gel electrophoresis; PBS: Phosphate-buffered saline; PDR: Proliferative diabetic retinopathy; pI: Isoelectric point; PMSF: Phenylmethylsulphonyl fluoride; PTM: Post-translational modification; Q: Quadrupole; RPC: Reverse phase chromatography; RPE: Retinal pigment epithelium; RPMI: Roswell Park Memorial Institute; RT-PCR: Reverse transcription polymerase chain reaction; SB3-10: Sulphobetaine 10; SCX: Strong cation exchange; SDS: Sodium dodecyl sulfate; SILAC: Stable isotope labelling with amino acids in cell culture; TBP: Tributylphosphine; TCA: Trichloroacetic acid; TM: Trabecular meshwork; TOF: Time-of-flight
